# A safe, effective, and reliable vaccine against Chagas disease should be described!

**DOI:** 10.1590/0074-02760210314chgsa

**Published:** 2022-05-09

**Authors:** João S Silva

**Affiliations:** 1 Fundação Oswaldo Cruz-Fiocruz

A safe, effective, and reliable vaccine against Chagas disease should be described!

I just read the wonderful review by Camargo et al.,^1^ ^)^ entitled “Why do we still have not a vaccine against Chagas disease?” and I strongly recommend reading it. The article brought me many incredible memories of the hot discussions during the Annual meeting on Chagas disease, in Caxambu, MG, Brazil. I first attended the meeting in 1979, and for the next three decades, I had the opportunity to take part in the discussions regarding the Chagas disease, the biology of *Trypanosoma cruzi*, its causal agent, and triatomines, the vector. At this time, it was well known the role of cellular and humoral immunity in the mediating resistance against the infection. Therefore, a robust antigenic stimulation of the immune system could result in the protection against the infection. As pointed in the review, diverse vaccination attempts with a variety of antigenic preparation (dead and attenuated parasites, cellular subfraction, purified native and recombinant proteins of epimastigotes and trypomastigotes, virus-based and DNA vaccines) were being tried to induce protection against the disease evaluated by parasitaemia, myocarditis, and mortality. Several experimental models have been used, including mice, rabbits, dogs, goats, rats, opossums, etc. It was very hard to convince the scientific community of the success of a particular antigenic preparation, even when the mortality rate and parasitaemia came to zero. The reasons were the inadequate experimental models, poor methods for parasite detection, added to the need to evaluate the protection against the different parasite genotypes (discrete typing units-DTUs, TcI-TcVI, and TcBat) and the well-known autoimmunity.

In the immunology sections, one of the hot topics was autoimmunity, the theory used to explain the neuronal destruction and the multifocal myocarditis that happens in chronic chagasic patients. There were no doubts regarding the phenomenon, but how to explain it. As the direct action of parasites (neurotoxic) had been discarded, it was almost unanimous that the autoimmune response raised against autoantigens or parasites antigens (antigenic mimicry) was responsible for the observed phenomena. Otherwise, how to explain the inflammatory infiltrate in the heart tissue in the apparent absence of parasites.

Publications showing peptides shared by the parasites and the heart tissue^2^ and syngeneic heart transplant rejection by infected mice,^3^ strongly suggested that autoimmunity was involved in the pathogenesis of chagasic cardiomyopathy in humans. The mechanism explaining the reduced ganglion cell number was only described in 2004,^4^ because of the IFN-gamma-elicited nitric oxide production close to neuronal ganglia. Summarising, the autoimmunity theory was currently accepted among the scientists, mainly after results showing myocarditis in mice after injections of autologous heart antigens.

In my view, the theory of autoimmunity was one strong reason that delayed and inhibited initiatives of research aimed at the development of vaccines against the disease. One other reason was the long-awaited sterilising immunity. Therefore, results showing protection against the infection, even in a complete absence of mortality, were severely criticised because a sterile immunity (not a “partialle immunité”) and a complete absence of myocarditis were always expected. In addition, the models for studying Chagas disease were (and they are) inadequate to prove that the postulated vaccine candidates were or not able to prevent the heart or digestive lesions in the chronic phase of infection (years later) and eventual parasite persistence in the tissues. Another major question was how to design a study to test the efficacy of vaccines on the prevention of the disease if it takes usually more than 10 years to appear (when it happens).

I am convinced that nowadays, with the incredible progress in omics technology, the availability of new adjuvants, and the knowledge of the immune system activation, it is possible to select “universal parasites antigens”, expressed in all seven DTUs, including in metacyclic, amastigote, and trypomastigote forms, that can induce antibody and CD8 T cell response, to protect against the disease. Using the new computational epitopes identification and vaccine design platform to define conserved parasite epitopes to develop antibody- and T-cell-directed vaccines, a therapeutic vaccine became an achievable task. Using mRNAs as therapeutic tools to the immunisation approach to protect against the disease is possible to induce protection. This promising immunisation strategy has been extensively studied against other infectious diseases and many cancer types.

We are not far from this dream. As pointed by Gazzinelli’s review,^1^ we already know the properties of an ideal vaccine to *T. cruzi* and they already have a vaccine able to protect mice from parasitaemia and mortality. We should remind that parasite elimination prevents the progression of the symptoms of Chagas disease^5^ and that the etiologic treatment is potentially useful to both the patients and public health. I hope that the scientists, using these available new technologies, can start new projects aiming to develop a vaccine against Chagas disease. Of course, it is not easy, but it is possible, and we deserve it to the community.

Comments on the article: Camargo EP, Gazzinelli RT, Morel CM, Precioso AR. Why do we still have not a vaccine against Chagas disease? Mem Inst Oswaldo Cruz. 2021; 116: e200314.
